# Cavity-mediated long-range interactions in levitated optomechanics

**DOI:** 10.1038/s41567-024-02405-3

**Published:** 2024-03-01

**Authors:** Jayadev Vijayan, Johannes Piotrowski, Carlos Gonzalez-Ballestero, Kevin Weber, Oriol Romero-Isart, Lukas Novotny

**Affiliations:** 1 Photonics Laboratory, ETH Zürich, Zürich, Switzerland; 2 Quantum Center, ETH Zürich, Zürich, Switzerland; 3 Institute for Quantum Optics and Quantum Information, Austrian Academy of Sciences, Innsbruck, Austria; 4 Institute for Theoretical Physics, University of Innsbruck, Innsbruck, Austria; 5 Present Address: Photon Science Institute, Department of Electrical and Electronic Engineering, University of Manchester, Manchester, UK; 6 Present Address: Institute for Theoretical Physics, Vienna University of Technology (TU Wien), Vienna, Austria

**Keywords:** Quantum mechanics, Quantum optics

## Abstract

The ability to engineer cavity-mediated interactions has emerged as a powerful tool for the generation of non-local correlations and the investigation of non-equilibrium phenomena in many-body systems. Levitated optomechanical systems have recently entered the multiparticle regime, which promises the use of arrays of strongly coupled massive oscillators to explore complex interacting systems and sensing. Here we demonstrate programmable cavity-mediated interactions between nanoparticles in vacuum by combining advances in multiparticle optical levitation and cavity-based quantum control. The interaction is mediated by photons scattered by spatially separated particles in a cavity, resulting in strong coupling that is long-range in nature. We investigate the scaling of the interaction strength with cavity detuning and interparticle separation and demonstrate the tunability of interactions between different mechanical modes. Our work will enable the exploration of many-body effects in nanoparticle arrays with programmable cavity-mediated interactions, generating entanglement of motion, and the use of interacting particle arrays for optomechanical sensing.

## Abstract (web summary)

Combining multiparticle levitation with cavity control enables cavity-mediated interaction between levitated nanoparticles, whose strength can be tailored via optical detuning and position of the two particles.

## Main

Exploring quantum physics at macroscopic scales is an exciting prospect, both for fundamental physics and developing technology^1–3^. However, in addition to the challenge of ground-state cooling massive objects, such endeavours require either large-scale delocalization of a single object or the entanglement of multiple objects. Levitodynamics, which deals with controlling the mechanical motion of massive oscillators in vacuum^2,4^, has made remarkable headway towards multiple-particle systems, with demonstrations of cooling^5^ and short-range coupling^6–11^ between nanoparticles levitated in free space. Furthermore, recent experiments with single particles in optical tweezers have established exquisite control over rotational dynamics^12–15^ and achieved quantum ground-state cooling of mechanical motion^16–21^. One of the next pivotal milestones towards macroscopic quantum physics is to entangle multiple particles via optical forces. However, this is not possible in free space as the entangling rate is not large enough to overcome the decoherence rates of the particles^22,23^. Therefore, it becomes desirable to use an optical cavity to mediate coupling between the particles.

Here we introduce such a capability to engineer programmable cavity-mediated interactions between multiple spatially separated particles in vacuum. The programmability arises from the use of acousto-optic deflectors (AODs) to generate tweezer arrays for trapping the particles^5,24^, which offer a high degree of control over parameters such as optical frequencies of the tweezers and cavity detuning, as well as mechanical frequencies and position of the particles. Such parameter control is crucial for precisely tuning the interaction strength and for choosing which particles and mechanical modes couple.

Most experimental systems that study many-body physics rely either on localized short-range interactions^25,26^ or a common cavity mode to mediate interactions^27,28^, and can only afford short-distance or all-to-all connectivity respectively. Recent experiments with superconducting qubits^29^ and cold atoms^30^ have managed to overcome this limitation and demonstrated tunability in the connectivity of interactions. In our platform, the decoupling of the trapping mechanism from the cavity presents the opportunity to engineer a broad range of connectivity, by programming specific tweezers to be resonant with the cavity mode. This new prospect in levitodynamics will facilitate progress towards generating quantum correlations and entanglement^22,31–36^, exploring complex phases emerging from interacting particles^26,37–39^ and using multiparticle quantum resources^40–43^ for optomechanical sensing^44–50^.

## Experimental setup

**Fig. 1:**
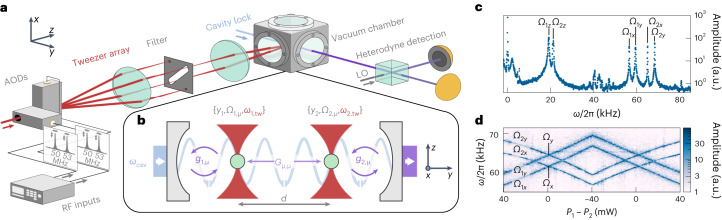
Cavity optomechanics with multiple nanoparticles. **a**, A laser beam is split using AODs and focused by a high-NA focusing lens inside a vacuum chamber to generate optical tweezers. The optical frequency of the tweezers *ω*_tw_, as well as the positions *y*_*i*_ of the particles (i ∈ (1, 2)) and the mechanical frequencies *Ω*_i,μ_ of their centre-of-mass modes (μ ∈ (*x*, *y*, *z*)), are controlled by programming the RF inputs of the AODs. **b**, The particles are positioned with a variable spatial separation *d* = ∣*y*_1_ − *y*_2_∣ along the standing wave of an optical cavity with resonant at *ω*_cav_, which is blue-detuned by *Δ* = *ω*_cav_ − *ω*_tw_. The individual optomechanical coupling of each particle with strength *g*_i,μ_ introduces an effective coupling *G*_μ,μ_ between the two particles. Tweezer light scattered by the particles leaks out of the cavity and is interfered with a local oscillator for balanced heterodyne detection. **c**, Spectrum of the heterodyne signal, where we set the Rayleigh peak frequency to *ω* = 0, showing the three mechanical anti-Stokes sidebands at *ω* = *Ω*_i,μ_ for each particle. **d**, Spectrogram of the anti-Stokes sidebands as a function of the power difference between the tweezers *P*_1_ − *P*_2_. Source data

The mechanical oscillators in our experiment comprise of near-spherical SiO_2_ nanoparticles with a nominal diameter of 150 nm, levitated in vacuum (~10^−4^ mbar) using optical tweezers (numerical aperture (NA) = 0.75) at wavelength *λ* = 1,550 nm (Fig. 1a). The particles are placed in the fundamental mode of an optical cavity with linewidth *κ*/2π = 600 kHz, comprised of mirrors with different transmissions separated by 9.6 mm (Fig. 1b). We use two tweezers with identical optical frequencies along the diagonal of a two-dimensional array of beams generated by two orthogonally placed AODs^51^. The cavity resonance is detuned by a frequency *Δ* with respect to both tweezers. Light scattered by the particles, carrying information about their centre-of-mass motion along the three axes *x*, *y* and *z*, leaks through the higher transmission cavity mirror. This light is combined with a local oscillator and split equally onto a balanced photodetector. The Fourier transform of the detector voltage gives the spectral amplitudes of our signal. For convenience, we offset the frequency axis to have the optical tweezer frequency at zero (Fig. 1c). The particle positions *y*_*i*_ (*i* ∈ (1, 2), labelling the particles) and their separation *d* = ∣*y*_1_ − *y*_2_∣ along the cavity axis are controlled by the frequencies sent to the radio-frequency (RF) channels of the AODs, while preserving the degeneracy of the tweezer optical frequencies (Methods, ‘Tweezer positioning and calibration’). In addition, a translation stage for the high-NA lens can displace both particles simultaneously. The spatial separation *d* between the nanoparticles (typically 6 μm) is large enough to suppress short-range Coulomb (∝ 1/*d*^3^) (refs. ^8,52^) and free-space optical binding (∝ 1/*d*) (ref. ^6^) interactions. The optical power of the tweezers *P*_*i*_ (typically 130 mW) and resulting mechanical frequencies *Ω*_*i*,*μ*_ (*μ* ∈ (*x*, *y*, *z*), labelling the mechanical modes) are set by the respective AOD RF amplitudes.

To engineer interactions in our experiments, we bring the mechanical frequencies of the two particles close together by carrying out linear ramps of the optical powers. We make use of coherent scattering, whereby light scattered off each nanoparticle populates the cavity mode and results in optomechanical coupling with strengths *g*_*i*,*μ*_ (ref. ^53^). This gives rise to effective cavity-mediated interactions between the nanoparticles. For particles in tweezers polarized along the cavity (*y*) axis, *g*_*i*,*μ*_ is zero and there is no particle–particle coupling (*G*_*μ*,*μ*_ ∝ *g*_1,*μ*_ *g*_2,*μ*_ = 0). Therefore, a power ramp simply results in crossings of the mechanical frequencies at *Ω*_*μ*_, as seen in the spectrogram in Fig. 1d. In the following, the tweezers are polarized along the *x* axis, maximizing light scattering along the cavity axis. The couplings along the *x* axis are minimal in this configuration, but the couplings along *y* and *z* axes can be maximal and result in cavity-mediated long-range interactions.

## Cavity-mediated long-range interactions

**Fig. 2:**
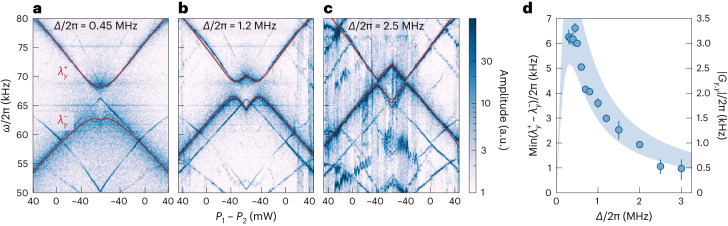
Cavity-mediated long-range interactions. **a**–**c**, Measured spectrograms show the normal mode splittings arising from cavity-mediated particle-particle interactions for different values of cavity detuning *Δ*. Red lines are fits of the normal mode frequencies ${\lambda }_{y}^{-}$ and ${\lambda }_{y}^{+}$ of the coupled system. **d**, The splitting $\min ({\lambda }_{y}^{{+}}-{\lambda }_{y}^{-})=2| {G}_{y,y}|$ of the avoided crossing extracted from the fits, as a function of detuning. Error bars correspond to three s.d. of the fit around the extracted splitting values. The shaded area shows theoretical estimations of the coupling strength *G*_*y*,*y*_ based on system parameters, exhibiting the characteristic dependence on cavity detuning. Source data

Two mechanical modes of different nanoparticles coupled to the same cavity mode via coherent scattering gives rise to an effective particle–particle coupling

1 $${G}_{\mu ,\mu }=\frac{{g}_{1,\mu }{({g}_{2,\mu })}^{* }}{\left(\varDelta +{\varOmega }_{\mu }\right)+\mathrm{i}\kappa /2}+\frac{{({g}_{1,\mu })}^{* }{g}_{2,\mu }}{\left(\varDelta -{\varOmega }_{\mu }\right)-\mathrm{i}\kappa /2},$$

when the mechanical frequencies *Ω*_1,*μ*_ ≈ *Ω*_2,*μ*_ are close to degeneracy (≈*Ω*_*μ*_). (Details are provided in Methods, ‘Coherent scattering with two particles’.) The structure of equation (1) is characteristic of cavity-mediated couplings in the fast-cavity regime (*κ* > *Ω*_*μ*_), and is formally analogous to the cavity-induced intraparticle couplings explored for single-particle coherent scattering^53–55^. In this work, we focus on configurations where the cavity can be treated as a bath and couples to only one mechanical mode per particle or to two far-detuned modes per particle, and thus the intraparticle couplings are negligible. Our coupled system has two normal modes with frequencies ${\lambda }_{\mu }^{-}$ and ${\lambda }_{\mu }^{+}$. The minimal splitting at the avoided crossing between these normal modes is $\min ({\lambda }_{\mu }^{{+}}-{\lambda }_{\mu }^{-})=2| {G}_{\mu ,\mu }|$. In Fig. 2a,b,c we show spectrograms of the *x* and *y* modes of two particles positioned at separate cavity nodes during power sweeps for *Δ*/2π = 0.45, 1.2, 2.5 MHz, respectively. As in Fig. 1d, the *x* modes cross at *Ω*_*x*_/2π ≈ 59 kHz, indicating no interactions of the *x* degree of freedom and offering a calibration of optical powers through ${\varOmega }_{i,x}\propto \sqrt{{P}_{i}}$. We fit expressions for ${\lambda }_{y}^{-}$ and ${\lambda }_{y}^{+}$ (Methods, ‘Extracting mode splitting’) to the normal modes in the spectrograms using the calibrated powers and the bare mechanical frequencies *Ω*_*i*,*y*_ at the edges of the ramp, with the only free parameter being the product of the individual optomechanical coupling strengths ∣*g*_1,*y*_ *g*_2,*y*_∣. We overlay the fits in Fig. 2a–c and extract $\min ({\lambda }_{y}^{{+}}-{\lambda }_{y}^{-})$, which follows the characteristic dependence on the detuning *Δ*, given by equation (1).

We see two other features of cavity optomechanics as the cavity is brought closer to resonance with the mechanical frequencies of the particles. First, the *y* modes of both particles are cooled via coherent scattering^21,56^ and become visibly broader in the spectrograms, whereas the *x* modes are not. Second, the optical spring effect^57^ causes a larger shift of the mechanical frequencies of the *y* modes as the detuning decreases. Therefore, the avoided crossings of the *y* modes appear shifted away from the crossings of the *x* modes at *P*_1_ = *P*_2_. Figure 2d shows the normal mode splitting over a wide range of detunings. The shaded area represents the particle-particle coupling strength calculated from equation (1), with *g*_1,*μ*_ and *g*_2,*μ*_ estimated from system parameters and their uncertainties (Methods, ‘Estimations of coupling strengths’). The measured splittings are in excellent agreement with the theoretical estimates. We observe a maximum splitting of $\min (\mathop{\lambda }\nolimits_{y}^{+}-\mathop{\lambda }\nolimits_{y}^{-})/2\pi =(6.6\pm 0.2)$ kHz at *Δ*/2π = 0.45 MHz, close to the optimal detuning.

## Distance-dependence of interactions

In addition to cavity-mediated interactions, the two particles can interact via Coulomb forces (provided they are charged) or via direct optical dipole-dipole coupling. Although such interactions offer a useful resource in levitated optomechanics—for example, for sympathetic cooling^7–9^, engineered coupling^6^ and synchronization^10^—they are of short-range nature (∝ 1/*d*^3^ and ∝ 1/*d* respectively). Furthermore, charged particles introduce electronic noise and the scattering losses of direct optical interactions prevent the generation of entanglement^23^.

**Fig. 3:**
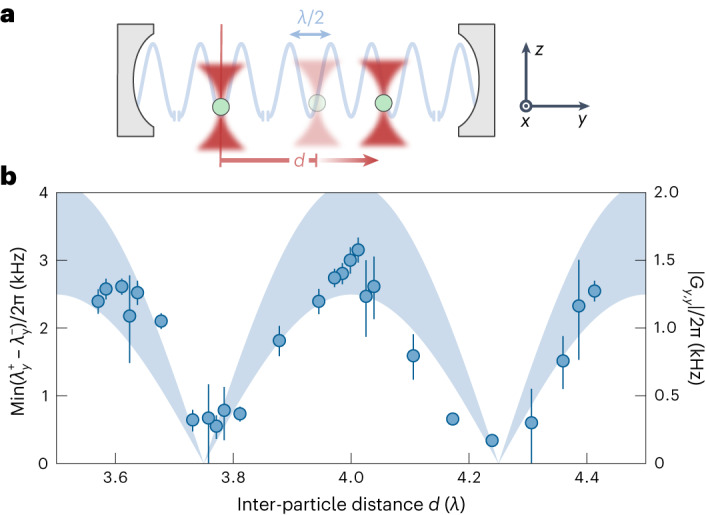
Distance-dependence of cavity-mediated interactions. **a**, Sketch of two particles placed in the standing wave of the cavity. The position of the first particle (left) is kept fixed at a node whereas the second particle (right) is moved along the standing wave, thereby increasing the interparticle distance *d*. **b**, Data points show the measured mode splitting as a function of *d* with error bars corresponding to three s.d. of the fit around the extracted splitting values. The shaded area shows the position dependence of ${g}_{2,y}\propto | \cos (2\uppi d/\lambda )|$ imprinted on the particle-particle coupling *G*_*y*,*y*_. Its width corresponds to uncertainties in system parameters. Source data

By examining the dependence on interparticle distance *d*, we show that the particle-particle interactions in our system are mediated via the cavity mode. We keep one particle stationary at a node and scan the position of the second particle along the nodes and antinodes of the cavity standing wave (Fig. 3a), while maintaining a fixed detuning of *Δ*/2π = 1.2 MHz. By briefly separating the optical frequencies of both tweezers and measuring the magnitude of the Rayleigh peaks in the heterodyne spectrum, we can independently determine the positions *y*_1_ and *y*_2_ of the two particles along the cavity standing wave (Methods, ‘Tweezer positioning and calibration’)^21,56^. We perform optical power sweeps, as in the previous section, for different distances and extract normal mode splittings from the resulting spectrograms. Figure 3b shows that as the position of the second particle is scanned along the cavity axis, the splitting exhibits a periodic behaviour. While the optomechanical coupling strength *g*_1,*y*_ is constant throughout this measurement (as *y*_1_ is fixed), *g*_2,*y*_ follows a periodic dependence on *d* (as we change *y*_2_) and imprints it on the particle–particle coupling through $| {G}_{y,y}| \propto | {g}_{2,y}| \propto | \cos (2\uppi d/\lambda )|$. The shaded area in Fig. 3b shows *G*_*y*,*y*_ as calculated from system parameters using equation (1), in agreement with the measured splittings. Conservative estimates of coupling strengths due to Coulomb (*G*_C_, 50 elementary charges on each nanoparticle) and short-range optical interactions (*G*_O_) from our system parameters at *d*/*λ* = 3.5 give maximal coupling values of *G*_C_/2π = 0.17 kHz and *G*_O_/2π = 0.14 kHz, respectively (Methods, ‘Estimations of coupling strengths’). Both are close to the resolution limit of our fitting procedure of ${G}_{\min }/2\uppi \approx 0.15$ kHz, given by the peak widths of 0.6 kHz.

## Tunability of interacting modes

The optomechanical coupling between each mode of the particle and the cavity is position dependent. The positioning control in our setup allows us to vary the relative interaction strengths of the transverse *y* mode and the longitudinal *z* mode. Previous studies have shown that the transverse coupling strengths scale as ${g}_{i,x},{g}_{i,y}\propto \sin {\varphi }_{i}$, and the longitudinal coupling strengths as ${g}_{i,z}\propto \cos {\varphi }_{i}$, where the phase factors *φ*_*i*_ encode the distance between the particle position *y*_*i*_ and the closest intensity maximum of the cavity^53,56^.

**Fig. 4:**
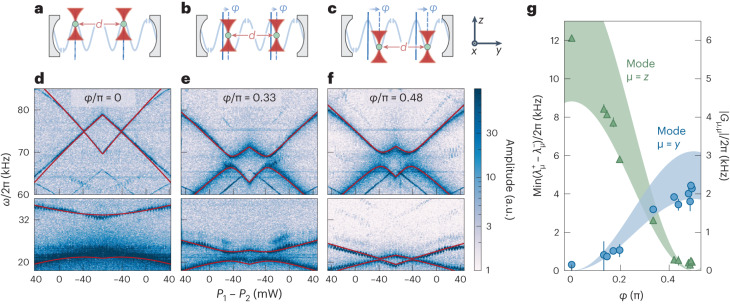
Tunability of interactions between mechanical modes. By keeping the interparticle separation *d* fixed and moving the particle pair along the cavity axis, we can tune the interaction strengths of different mechanical modes. The distance of each particle to the closest cavity node is characterized by the phase factor *φ*. **a**, Both particles are located at antinodes. **d**, The *z* modes interact giving rise to substantial mode splitting, whereas the *y* modes show no sign of interactions (no mode splitting). **b**, Both particles are positioned along slopes of the standing wave. **e**, The *z* mode splitting decreases and the *y* modes start splitting. **c**, Both particles are located at nodes. **f**, The *y* modes show avoided crossing, whereas the splitting of the *z* modes fades away. In all spectrograms, we fit the normal mode frequencies ${\lambda }_{y}^{-}$ and ${\lambda }_{y}^{+}$ (red lines). **g**, The splittings $\min ({\lambda }_{z}^{{+}}-{\lambda }_{z}^{-})$ (green triangles) and $\min ({\lambda }_{y}^{{+}}-{\lambda }_{y}^{-})$ (blue circles) as extracted from the fits with error bars corresponding to three s.d. of the fit around the extracted splitting values. Strong *z* coupling at the antinodes (*φ* = 0) transitions to strong *y* coupling at the nodes (*φ* = π/2). Shaded areas show *G*_*z*,*z*_ (green) and *G*_*y*,*y*_ (blue) from system parameters and their uncertainties. Source data

In our final experiment, we keep the interparticle separation fixed at *d* = 4*λ*, such that *φ*_1_ = *φ*_2_ = *φ*, and move both particles simultaneously along the standing wave from antinodes (*φ* = 0) to nodes (*φ* = π/2) (Fig. 4a–c). Doing so, we observe the cavity-mediated interactions transition from the *z* modes to the *y* modes (Fig. 4d–f). Notably, at antinodes (Fig. 4d), we observe a large normal mode splitting for the *z* mode, corresponding to particle-particle coupling as high as *G*_*zz*_/*Ω*_*z*_ = 0.238 ± 0.005. The mode splittings as a function of phase between *φ* = 0 and *φ* = *π*/2 are shown in Fig. 4g. The shaded areas are the particle-particle coupling strengths estimated from system parameters and their uncertainties, exhibiting the dependencies ${G}_{y,y}\propto {\sin }^{2}\varphi$ and ${G}_{z,z}\propto {\cos }^{2}\varphi$. Therefore, the choice of the position of our particles allows us to precisely tune the relative interaction strengths of the different modes of the mechanical oscillator.

## Conclusions

Combining the capabilities of cavity-based coherent scattering with multiparticle levitation provides a new platform for optomechanics. The high degree of control over parameters such as cavity detuning, mechanical frequency, polarization and particle position has allowed us to engineer and investigate the nature of cavity-mediated long-range interactions between two mechanical oscillators. We investigated the scaling of the interactions strength of the transverse *y* modes of two particles with cavity detuning, explored the distance-dependence of the interactions and finally showed that we can tune the interactions of different mechanical modes of the two particles. The highest interaction strength we report is *G*_zz_/*Ω*_z_ = 0.238 ± 0.005 for the longitudinal *z* modes. This value is higher than reported in free-space experiments^6^, despite the much larger separations in our experiments. Our scheme can be readily scaled up to more particles^5^ and brought into the quantum regime by increasing trap frequencies using a higher-power laser and by lowering the pressure by baking the chamber. Additionally, switching to a cavity with smaller mode volume or a narrower linewidth^57,58^ can increase the interaction strength further to meet the requirements for motional entanglement^22^.

Ultimately, the ability to engineer programmable cavity-mediated interactions between levitated systems offers a powerful new resource in optomechanics. Together with advances in achieving quantum control of mechanical motion and scaling up to nanoparticle arrays, our work firmly establishes levitodynamics as a compelling platform to explore the boundaries of quantum physics with massive interacting mechanical systems, and to build ultra-precise sensors with optomechanical arrays.

## Methods

### Tweezer array generation

**Extended Data Fig. 1:**
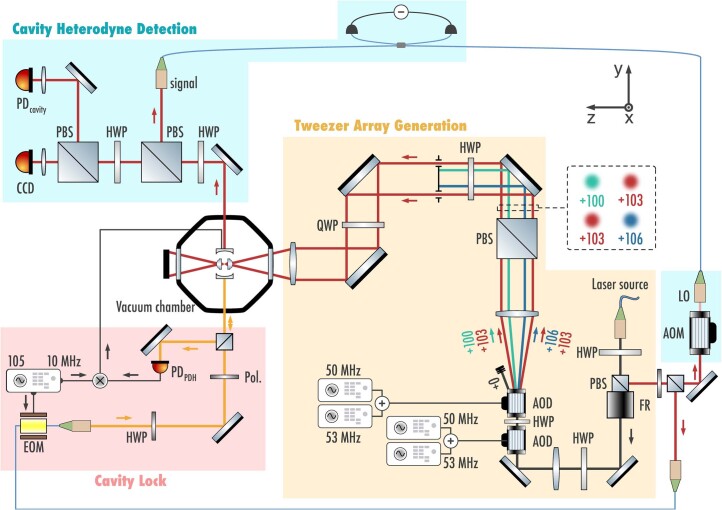
Sketch of full experimental setup. Tweezer arrays are generated using two AODs fed by two RF input channels each (orange box). Two beams with the same optical frequency are sent into the vacuum chamber to trap particles. A Pound–Drever–Hall scheme is used to lock the cavity (red box), and a balanced heterodyne detection scheme is used to obtain spectral information about mechanical motion (blue box).

A complete sketch of the setup used in this study is shown in Extended Data Fig. 1. The orange box indicates the part of the setup used in the generation of tweezer arrays. Laser light at 1,550 nm is sent through two AODs (AA optoelectronics DSTX), that are placed orthogonal to one another. Each AOD receives the sum of two RF frequencies close to its central frequency, generally one at 50 MHz and the other at 53 MHz, generated by two different channels of a function generator (MOGLABS Agile RF Synthesizer). As a result, four optical beams with frequencies 100 MHz, 103 MHz, 103 MHz and 106 MHz are generated in the first order following both AODs (see inset of Extended Data Fig. 1). A spatial filter is then used to remove the 100 MHz and 106 MHz beams, along with any other zero order beams, resulting in only the two diagonal beams at 103 MHz making it to the high-NA lens inside the chamber. We load our particles by dispersing them in solution near the tweezers while mechanically shielding the cavity mirrors to avoid contamination. The two orthogonal AODs are mounted at an angle of 45° with respect to the optical table, ensuring that the two 103 MHz beams are parallel to the cavity. The RF amplitudes of all four channels are normally kept at 32 dBm. The RF frequencies and amplitudes are fully programmable, a feature that is heavily used in our experiments—the tunability of the frequency allows us to position the particles along the cavity, and the tunability of the amplitude allows us to perform sweeps of their motional frequencies.

### Tweezer positioning and calibration

In our investigation of distance-dependence of the interactions, we change the interparticle separation while keeping the tweezers at the same frequency. The frequencies of one RF channel of both tweezers are increased (decreased), leading to both tweezers moving further apart (closer together). We then move both particles common-mode using a linear translation stage (Attocube ANP(*x*,*y*,*z*)101) to bring particle 1 back to its initial position, in this case a node of the cavity. To confirm the position at the node we momentarily introduce a frequency difference of 1 kHz between the two tweezers to separate their signatures in the spectrum and move the Attocube stage, if required, to minimize the Rayleigh scattered light from particle 1. Finally, the cavity detuning is adjusted to the new optical frequency common to both tweezers. Repeating these steps, we change the interparticle distance while keeping one particle at a node and the detuning fixed. This procedure also shifts both particles perpendicular to the cavity axis. We can neglect this effect as the displacements of few micrometres are far smaller than the cavity waist *W*_c_ = 50 μm. Alternatively, if we use both channels of the AODs that go to each tweezer, we can move both particles only along the cavity axis, or only perpendicular to it while changing the tweezers frequency. Any changes to the tweezers’ positions by adjusting the RF frequencies or moving the translation stage are negligibly small compared to the open aperture of our trapping lens of about 1 mm and do not affect the trapping frequencies.

**Extended Data Fig. 2:**
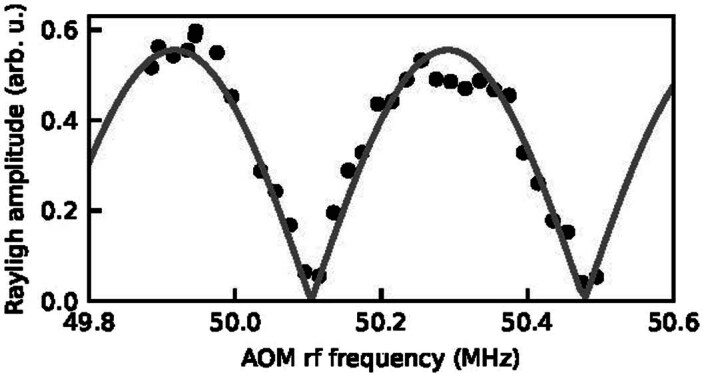
Calibration of RF to displacement. The periodicity of measured carrier amplitude extracted by a fit (line) gives the conversion factor between RF and particle displacement. Source data

To calibrate the spatial separation, we sweep the input RF and observe the height of the Rayleigh peak of a single particle on the heterodyne detector. Extended Data Fig. 2 shows the Rayleigh amplitude ${I}_{{{{\rm{RL}}}}}\propto | \cos (\varphi )|$ scales with the position *φ* in the standing wave, which is periodic with *λ*/2. By extracting the periodicity of the fit (line) to the data points we get the conversion factor (1.34 ± 0.02)*λ*/MHz of RF to displacement. Since the tweezers’ position scales linearly with the RF applied to the AODs, we can use this conversion factor to obtain the usual interparticle distance for RF inputs of 50 MHz and 53 MHz of (4.01 ± 0.05)*λ* ≈ 6 μm.

### Coherent scattering with two nanoparticles

The theory for coherent scattering of a single nanoparticle can be found in ref. ^53^. Here we briefly lay out the necessary extensions for two nanoparticles and the emerging effective coupling. We consider two particles each trapped by a different optical tweezers and coupled to an optical cavity. We assume both lasers have the same frequency *ω*_0_ and waist *W*_t_, and are polarized either parallel or perpendicular to the cavity axis *y*, propagate along *z*, and that their foci are separated by a distance *d* ≫ *W*_t_. The dynamics of the system formed by the single cavity mode and the centre-of-mass motion of the two particles is governed by the following master equation for its density matrix, $\hat{\rho }$:

2 $$\begin{array}{rcl}\dot{\hat{\rho }}&=&-\frac{\rm{i}}{\hslash }\left[\hat{H},\hat{\rho }\right]+\kappa \left(\hat{c}\hat{\rho }{\hat{c}}^{{\dagger} }-\frac{1}{2}\left\{{\hat{c}}^{{\dagger} }\hat{c},\hat{\rho }\right\}\right)\\ &&-\mathop{\sum}\limits_{i=1,2}\mathop{\sum}\limits_{\mu =x,y,z}\frac{{\varGamma }_{i\mu }}{2}\left[{\hat{b}}_{i\mu }+{\hat{b}}_{i\mu }^{{\dagger} },\left[{\hat{b}}_{i\mu }+{\hat{b}}_{i\mu }^{{\dagger} },\hat{\rho }\right]\right]\\ &&+\mathop{\sum}\limits_{i=1,2}\mathop{\sum}\limits_{\mu =x,y,z}\frac{{\gamma }_{i\mu }}{4}\left[{\hat{b}}_{i\mu }+{\hat{b}}_{i\mu }^{{\dagger} },\left\{{\hat{b}}_{i\mu }^{{\dagger} }-{\hat{b}}_{i\mu },\hat{\rho }\right\}\right]\end{array}$$

where *κ* is the cavity linewidth, $\hat{c}$ is the annihilation operator of a cavity photon and the curly brackets denote the anticommutator. The motional mode *μ* of particle *i* is characterized by an annihilation operator ${\hat{b}}_{i\mu }$, a friction rate *γ*_*iμ*_ and a heating rate *Γ*_*iμ*_ that includes contributions from surrounding gas molecules and from laser shot noise. In a frame rotating at the frequency of the two optical tweezers *ω*_0_ the coherent scattering Hamiltonian is

3 $$\begin{array}{rcl}\hat{H}&=&\hslash \mathop{\sum}\limits_{i=1,2}\mathop{\sum}\limits_{\mu =x,y,z}{\varOmega }_{i,\mu }{\hat{b}}_{i\mu }^{{\dagger} }{\hat{b}}_{i\mu }\\ &&+\hslash \varDelta {\hat{c}}^{{\dagger} }\hat{c}+\hslash \left({\varOmega }_{c}{\hat{c}}^{{\dagger} }+{\varOmega }_{c}^{* }\hat{c}\right)\\ &&+\hslash \mathop{\sum}\limits_{i}\mathop{\sum}\limits_{\mu }\left[{\hat{c}}^{{\dagger} }{g}_{i\mu }\left({\hat{b}}_{i\mu }^{{\dagger} }+{\hat{b}}_{i\mu }\right)+\,{{\mbox{H.c.}}}\,\right],\end{array}$$

with *Δ* = *ω*_c_ − *ω*_0_, *g*_*α*_ the coherent scattering couplings, H.c. the Hermitian conjugate and a cavity drive at frequency *ω*_c_ given by

4 $${\varOmega }_{{\mathrm{c}}}= -\frac{1}{2}\sqrt{\frac{{\omega }_{{\mathrm{c}}}}{2\hslash {\varepsilon }_{0}{V}_{{\mathrm{c}}}}}\mathop{\sum}\limits_{j}{\alpha }_{j}{E}_{0j}\cos {\theta }_{j}\cos {\varphi }_{j}{e}^{-\mathrm{i}{\Phi }_{j}}.$$

Here, *ω*_c_ and *V*_c_ are the cavity bare frequency and mode volume, *α*_*j*_ is the polarizability of particle *j*, *E*_0*j*_ and *θ*_*j*_ the electric field amplitude and polarization angle of tweezer *j* at its focus (*θ*_*j*_ = 0 for polarization perpendicular to cavity axis, *θ*_*j*_ = π/2 for polarization along cavity axis), and *Φ*_*j*_ the phase of each trapping laser, which we choose for convention as *Φ*_1_ = 0, *Φ*_2_ = *Φ*. The angle *φ*_*j*_ encodes the position of the focus of tweezer *j* within the cavity mode profile, specifically *φ*_*j*_ = 0 at an antinode and *φ*_*j*_ = π/2 at a node. For ∣*Ω*_c_/*κ*∣ ≪ 1 the coherent scattering couplings read

5 $$\begin{array}{rcl}\left[\begin{array}{c}{g}_{jx}\\ {g}_{jy}\\ {g}_{jz}\end{array}\right]&=&\sqrt{\frac{{\omega }_{{\mathrm{c}}}}{2\hslash {\epsilon }_{0}{V}_{{\mathrm{c}}}}}\frac{{\alpha }_{j}{E}_{0j}}{2}\cos \left({\theta }_{j}\right){e}^{-\mathrm{i}{\Phi }_{j}}\\ &\times &\left[\begin{array}{c}{(-1)}^{\;j}{k}_{{\mathrm{c}}}{x}_{0j}\sin {\varphi }_{j}\sin {\theta }_{j}\\ {(-1)}^{\;j}{k}_{{\mathrm{c}}}{\;y}_{0j}\sin {\varphi }_{j}\cos {\theta }_{j}\\ -\mathrm{i}{k}_{0}{z}_{0j}\cos {\varphi }_{j}\end{array}\right]\end{array},$$

with *k*_c_ and *k*_0_ the wavenumber of cavity and tweezer modes and $\left\{{x}_{0j},{y}_{0j},{z}_{0j}\right\}$ the zero-point motion along the three axes. This expression also assumes that the cavity Rayleigh range is much larger than the separation between particles, which is the case for this experiment as ${y}_{{{{\rm{R}}}}}=\uppi {W}_{{{{\rm{c}}}}}^{\;2}/{\lambda }_{0}=(5\pm 1)$ mm ≫ *d* (with *W*_c_ the cavity waist).

To compute the eigenfrequencies of the coupled system, that is the peaks of the cavity power spectral density, it is sufficient to compute the dynamics of the classical motional amplitudes, defined by the vector

6 $${{{\bf{v}}}}\equiv \left[\left\langle {\hat{b}}_{1x}\right\rangle ,\left\langle {\hat{b}}_{1y}\right\rangle ,\left\langle {\hat{b}}_{1z}\right\rangle ,\left\langle {\hat{b}}_{2x}\right\rangle ,\left\langle {\hat{b}}_{2y}\right\rangle ,\left\langle {\hat{b}}_{2z}\right\rangle \right].$$

We compute their dynamics by first adiabatically eliminating the cavity^59,60^ to obtain a reduced master equation for the motional modes. This procedure is valid when *κ* ≫ ∣*g*_*iμ*_, *γ*_*iμ*_∣ and amounts to considering the cavity a passive bath that couples the motional modes. Then, we use the resulting master equation to compute the dynamics of the vector of mechanical amplitudes, given in the underdamped regime ∣*γ*_*iμ*_∣ ≪ *Ω*_*i*,*μ*_ by

7 $$\frac{{\mathrm{d}}}{{\mathrm{d}}t}{{{\bf{v}}}}=-\mathrm{i}\bar{A}{{{\bf{v}}}},$$

with a dynamical matrix

8 $${\bar{A}}_{\alpha {\alpha }^{{\prime} }}\equiv \left[{\varOmega }_{\alpha }-\mathrm{i}\frac{{\gamma }_{\alpha }}{2}\right]{\delta }_{\alpha {\alpha }^{{\prime} }}+{G}_{\alpha {\alpha }^{{\prime} }}\,,$$

where we defined the multi-index *α* = {*j* = (1, 2), *μ* = (*x*, *y*, *z*)}. The effective cavity-mediated couplings read

9 $${G}_{\alpha {\alpha }^{{\prime} }}=\frac{{g}_{{\alpha }^{{\prime} }}^{\;* }{g}_{\alpha }}{\left(\varDelta +{\varOmega }_{{\alpha }^{{\prime} }}\right)+\mathrm{i}\kappa /2}+\frac{{g}_{{\alpha }^{{\prime} }}{g}_{\alpha }^{\;* }}{\left(\varDelta -{\varOmega }_{{\alpha }^{{\prime} }}\right)+\mathrm{i}\kappa /2}\,.$$

Note that aside from couplings between different modes the cavity also induces a mechanical frequency shift *G*_*αα*_ in mode *α*. Note also that the couplings are in general non-reciprocal, that is, ${G}_{\alpha {\alpha }^{{\prime} }}\ne {G}_{{\alpha }^{{\prime} }\alpha }$.

The peaks of the spectrum will be centred at frequencies ${{\mathrm{Re}}}\,\left[{\Lambda }_{l}\right]$ and have linewidth ${{\mathrm{Im}}}\,\left[{\Lambda }_{l}\right]$, where *Λ*_*l*_ are the six eigenvalues of the matrix $\bar{A}$. All mechanical modes that do not couple to the cavity (*g*_*α*_ = 0) remain uncoupled in this effective picture and give rise to trivial eigenvalues *Λ*_*l*_ = *Ω*_*α*_ − i*γ*_*α*_/2. In the experiment we consider purely *x-*polarized tweezers so that both *x* mechanical modes are uncoupled from the cavity. If we particularize to the case where both particles are at the node (*φ*_1_ = *φ*_2_ = π/2) or at the antinode (*φ*_1_ = *φ*_2_ = π) two more mechanical modes are uncoupled from the cavity, namely the *y* modes at the node and the *z* modes at the antinode. In these cases the remaining two mechanical modes *α* = {1, *μ*} and ${\alpha }^{{\prime} }=\{2,\mu \}$ form a 2 × 2 coupled system which can be diagonalized analytically to obtain the following two eigenvalues,

10 $$\begin{array}{rlr}{\varLambda }_{\mu }^{\pm }=\frac{1}{2}&\bigg[{D}_{1}(\varDelta )+{D}_{2}(\varDelta )&\\ &\pm \sqrt{{\left({D}_{1}(\varDelta )-{D}_{2}(\varDelta )\right)}^{2}+4{G}_{1\mu 2\mu }{G}_{2\mu 1\mu }}\bigg]\end{array}$$

with

11 $${D}_{j}(\varDelta )\equiv {\varOmega }_{j,\mu }-\mathrm{i}\frac{{\gamma }_{j\mu }}{2}+{G}_{j\mu j\mu }.$$

The normal mode frequencies ${\lambda }_{\mu }^{\pm }$ correspond to the real part of ${\Lambda }_{\mu }^{\pm }$, and their difference corresponds to the mode anticrossing

12 $${\lambda }_{\mu }^{+}-{\lambda }_{\mu }^{-}={{\mathrm{Re}}}\,\sqrt{{\left({D}_{1}(\varDelta )-{D}_{2}(\varDelta )\right)}^{2}+4{G}_{1\mu 2\mu }{G}_{2\mu 1\mu }}.$$

At the point of avoided crossing, where *Ω*_1,*μ*_ = *Ω*_2,*μ*_, for identical particles, and for in-phase tweezers (*Φ*_2_ = 0), the cavity-mediated couplings become reciprocal and the anticrossing simplifies to $2\sqrt{{G}_{1\mu ,2\mu }{G}_{2\mu ,1\mu }}\approx 2| {G}_{1\mu ,2\mu }| \equiv 2| {G}_{\mu ,\mu }|$ as used in the main text.

### Extracting mode splitting

To extract the mode splittings $\min (| \mathop{\lambda }\nolimits_{\mu }^{+}-\mathop{\lambda }\nolimits_{\mu }^{-}| )$ from the shapes in our measured spectrograms, we use equation (12). The normal mode frequencies are dependent on cavity parameters *κ* and *Δ*, which we independently measure, the power *P*_*i*_ in each of the tweezers, and the bare mechanical frequencies *Ω*_*i*,*μ*_. First, we run a peak finding algorithm for each slice of the spectrograms and sort the peaks into their respective modes. We then extract *Ω*_*i*,*μ*_ from the edges of the spectrograms. The uncoupled *x* peaks give a calibration for the relative powers by fitting a square-root function (${P}_{i}\propto \sqrt{{\varOmega }_{i,x}}$) to them. Finally, we subtract the peaks corresponding to the modes we want to fit from each other and fit equation (12), inserting the cavity parameters, powers and bare frequencies. The only fit parameter left is the product of optomechanical couplings *g*_1,*μ*_*g*_2,*μ*_. The mode splittings are then presented as the minimal separation between the fitted lines $\min (| {\lambda }_{\mu }^{+}-{\lambda }_{\mu }^{-}| )$. For strongly coupled modes (for example in Fig. 4d where *G*_*zz*_/*Ω*_*z*_ = 0.24) the fidelity of the fit is reduced. The strong coupling case and the cooling of such strongly hybridized modes will be the topic of a future study.

### Estimations of coupling strengths

For all estimations of coupling strengths, we use the system parameters in Extended Data Table 1. *Δ* and *κ* are taken from the main text and we set *φ* via from the position in the cavity. From our parameters and with equation (5) we estimate maximal optomechanical coupling strengths of *g*_*i*,*y*_/2π = (32 ± 4) kHz and *g*_*i*,*z*_/2π = (50 ± 7) kHz for *φ* = π/2 and *φ* = 0, respectively. We then get the effective cavity-mediated couplings calculated from equation (9) and present them including uncertainties in the system parameters as shaded areas in all plots.

Using the same system parameters, we estimate the coupling strengths of direct optical and Coulomb interactions from

13 $$\begin{array}{rcl}| {G}_{{{{\rm{OB}}}}}| &=&\frac{{\alpha }^{2}{k}_{0}^{5}{P}_{{{{\rm{t}}}}}}{4c{W}_{{{{\rm{t}}}}}^{\;2}{\uppi }^{2}{\epsilon }_{0}^{2}m\varOmega }\frac{\cos \left({k}_{0}d\right)}{k0d}\,,\\ | {G}_{{{{\rm{C}}}}}| &=&\frac{{Q}_{1}{Q}_{2}}{8\uppi {\epsilon }_{0}{d}^{\;3}}\frac{1}{m\sqrt{{\varOmega }_{1}{\varOmega }_{2}}}\,.\end{array}$$

We make the conservative assumption of *Q*_1_ = *Q*_2_ = 50*e* as nanoparticles of our size, loaded by spraying a fine solution into the trapping volume, typically hold few tens of elementary charges. For the minimum interparticle distance of *d*/*λ* = 3.5 in Fig. 3 we obtain *G*_C_/2π = (0.17 ± 0.03) kHz and *G*_O_/2π = (0.14 ± 0.04) kHz, respectively. Typical peak widths broadened by cavity cooling in our experiments are (0.6 ± 0.1) kHz. Assuming we can resolve peaks separated by half their widths, we estimate a minimum measurable coupling strength of ${G}_{\min }/2\pi \approx 0.15$ kHz close to both *G*_C_ and *G*_O_.

## Online content

Any methods, additional references, Nature Portfolio reporting summaries, source data, extended data, supplementary information, acknowledgements, peer review information; details of author contributions and competing interests; and statements of data and code availability are available at 10.1038/s41567-024-02405-3.

### Source data

Source Data Fig. 1 Source data for Figs.1c,d.

Source Data Fig. 2 Source data for Fig. 2.

Source Data Fig. 3 Source data for Fig. 3b.

Source Data Fig. 4 Source data for Figs. d-f,g.

Source Data Extended Data Fig. 2 Source data for Extended Data Fig. 2.

## Figures and Tables

**Extended Data Table 1:**
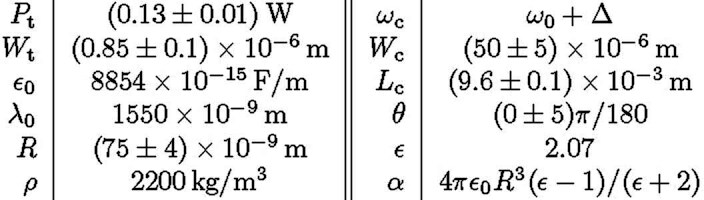
Experimental parameters and their uncertainties used for estimating coupling rates

P_t_: optical powers of both tweezers at the avoided crossing, W_t_: waist of the tweezers, R: nominal radius of the nanoparticles, *ρ*: density of the nanoparticles, *ω*_c_: frequency of the cavity field, *W*_c_: cavity waist, *L*_c_: cavity length, *ϵ*: electrical susceptibility of the nanoparticles, *α*: polarizability of the nanoparticles.

## Data Availability

Source data are provided with this paper and are available via the ETH Zurich Research Collection (10.3929/ethz-b-000653191). All other data that support the plots within this paper and other findings of this study are available from the corresponding author upon reasonable request.

## References

[1] Leggett AJ (2002). Testing the limits of quantum mechanics: motivation, state of play, prospects. J. Phys. Condens. Matter.

[2] Gonzalez-Ballestero C, Aspelmeyer M, Novotny L, Quidant R, Romero-Isart O (2021). Levitodynamics: levitation and control of microscopic objects in vacuum. Science.

[3] Rademacher M, Millen J, Li YL (2020). Quantum sensing with nanoparticles for gravimetry: when bigger is better. Adv. Opt. Technol..

[4] Millen J, Monteiro TS, Pettit R, Vamivakas AN (2020). Optomechanics with levitated particles. Rep. Prog. Phys..

[5] Vijayan J (2023). Scalable all-optical cold damping of levitated nanoparticles. Nat. Nanotechnol..

[6] Rieser J (2022). Tunable light-induced dipole-dipole interaction between optically levitated nanoparticles. Science.

[7] Arita Y (2022). All-optical sub-kelvin sympathetic cooling of a levitated microsphere in vacuum. Optica.

[8] Penny TW, Pontin A, Barker PF (2023). Sympathetic cooling and squeezing of two colevitated nanoparticles. Phys. Rev. Res..

[9] Bykov DS, Dania L, Goschin F, Northup TE (2023). 3d sympathetic cooling and detection of levitated nanoparticles. Optica.

[10] Brzobohatý O (2023). Synchronization of spin-driven limit cycle oscillators optically levitated in vacuum. Nat. Commun..

[11] Liška V (2023). Cold damping of levitated optically coupled nanoparticles. Optica.

[12] Bang J (2020). Five-dimensional cooling and nonlinear dynamics of an optically levitated nanodumbbell. Phys. Rev. Res..

[13] van der Laan F (2021). Sub-kelvin feedback cooling and heating dynamics of an optically levitated librator. Phys. Rev. Lett..

[14] Pontin A, Fu H, Toroš M, Monteiro TS, Barker PF (2023). Simultaneous cavity cooling of all six degrees of freedom of a levitated nanoparticle. Nat. Phys..

[15] Zielińska JA (2023). Controlling optomechanical libration with the degree of polarization. Phys. Rev. Lett..

[16] Delić U (2020). Cooling of a levitated nanoparticle to the motional quantum ground state. Science.

[17] Magrini L (2021). Real-time optimal quantum control of mechanical motion at room temperature. Nature.

[18] Tebbenjohanns F, Mattana ML, Rossi M, Frimmer M, Novotny L (2021). Quantum control of a nanoparticle optically levitated in cryogenic free space. Nature.

[19] Kamba M, Shimizu R, Aikawa K (2022). Optical cold damping of neutral nanoparticles near the ground state in an optical lattice. Opt. Express.

[20] Ranfagni A, Børkje K, Marino F, Marin F (2022). Two-dimensional quantum motion of a levitated nanosphere. Phys. Rev. Res..

[21] Piotrowski J (2023). Simultaneous ground-state cooling of two mechanical modes of a levitated nanoparticle. Nat. Phys..

[22] Rudolph H, Hornberger K, Stickler BA (2020). Entangling levitated nanoparticles by coherent scattering. Phys. Rev. A.

[23] Rudolph, H., Delić, U., Hornberger, K. & Stickler, B. A. Quantum theory of non-hermitian optical binding between nanoparticles. Preprint at arXiv:2306.11893 [quant-ph] (2023).

[24] Yan J, Yu X, Han ZV, Li T, Zhang J (2023). On-demand assembly of optically levitated nanoparticle arrays in vacuum. Photonics Res..

[25] Jurcevic P (2014). Quasiparticle engineering and entanglement propagation in a quantum many-body system. Nature.

[26] Bernien H (2017). Probing many-body dynamics on a 51-atom quantum simulator. Nature.

[27] Leroux ID, Schleier-Smith MH, Vuletić V (2010). Implementation of cavity squeezing of a collective atomic spin. Phys. Rev. Lett..

[28] Pedrozo-Peñafiel E (2020). Entanglement on an optical atomic-clock transition. Nature.

[29] Kollár AJ, Fitzpatrick M, Houck AA (2019). Hyperbolic lattices in circuit quantum electrodynamics. Nature.

[30] Periwal A (2021). Programmable interactions and emergent geometry in an array of atom clouds. Nature.

[31] Chauhan AK, Černotík O, Filip R (2020). Stationary gaussian entanglement between levitated nanoparticles. New J. Phys..

[32] Brandão I, Tandeitnik D, Guerreiro T (2021). Coherent scattering-mediated correlations between levitated nanospheres. Quantum Sci. Technol..

[33] Kotler S (2021). Direct observation of deterministic macroscopic entanglement. Science.

[34] de Lépinay LM, Ockeloen-Korppi CF, Woolley MJ, Sillanpää MA (2021). Quantum mechanics-free subsystem with mechanical oscillators. Science.

[35] Weiss T, Roda-Llordes M, Torrontegui E, Aspelmeyer M, Romero-Isart O (2021). Large quantum delocalization of a levitated nanoparticle using optimal control: applications for force sensing and entangling via weak forces. Phys. Rev. Lett..

[36] Chauhan AK, Černotík O, Filip R (2022). Tuneable gaussian entanglement in levitated nanoparticle arrays. Npj Quantum Inf..

[37] Reimann R (2015). Cavity-modified collective rayleigh scattering of two atoms. Phys. Rev. Lett..

[38] Landig R (2016). Quantum phases from competing short- and long-range interactions in an optical lattice. Nature.

[39] Liu S, Yin Z-q, Li T (2020). Prethermalization and nonreciprocal phonon transport in a levitated optomechanical array. Adv. Quantum Technol..

[40] Giovannetti V, Lloyd S, Maccone L (2004). Quantum-enhanced measurements: beating the standard quantum limit. Science.

[41] Demkowicz-Dobrzański R, Kołodyński J, GuŢă M (2012). The elusive heisenberg limit in quantum-enhanced metrology. Nat. Commun..

[42] Gessner M, Pezzè L, Smerzi A (2018). Sensitivity bounds for multiparameter quantum metrology. Phys. Rev. Lett..

[43] Pezzè L, Smerzi A, Oberthaler MK, Schmied R, Treutlein P (2018). Quantum metrology with nonclassical states of atomic ensembles. Rev. Mod. Phys..

[44] Ranjit G, Cunningham M, Casey K, Geraci AA (2016). Zeptonewton force sensing with nanospheres in an optical lattice. Phys. Rev. A.

[45] Jackson Kimball DF, Sushkov AO, Budker D (2016). Precessing ferromagnetic needle magnetometer. Phys. Rev. Lett..

[46] Hempston D (2017). Force sensing with an optically levitated charged nanoparticle. Appl. Phys. Lett..

[47] Monteiro F (2020). Search for composite dark matter with optically levitated sensors. Phys. Rev. Lett..

[48] Monteiro F (2020). Force and acceleration sensing with optically levitated nanogram masses at microkelvin temperatures. Phys. Rev. A.

[49] Carney D, Ghosh S, Krnjaic G, Taylor JM (2020). Proposal for gravitational direct detection of dark matter. Phys. Rev. D.

[50] Brady AJ (2023). Entanglement-enhanced optomechanical sensor array with application to dark matter searches. Commun. Phys..

[51] Endres M (2016). Atom-by-atom assembly of defect-free one-dimensional cold atom arrays. Science.

[52] Rudolph H, Delić U, Aspelmeyer M, Hornberger K, Stickler BA (2022). Force-gradient sensing and entanglement via feedback cooling of interacting nanoparticles. Phys. Rev. Lett..

[53] Gonzalez-Ballestero C (2019). Theory for cavity cooling of levitated nanoparticles via coherent scattering: master equation approach. Phys. Rev. A.

[54] Toroš M, Monteiro TS (2020). Quantum sensing and cooling in three-dimensional levitated cavity optomechanics. Phys. Rev. Res..

[55] Pontin A, Fu H, Iacoponi JH, Barker PF, Monteiro TS (2023). Controlling mode orientations and frequencies in levitated cavity optomechanics. Phys. Rev. Res..

[56] Windey D (2019). Cavity-based 3d cooling of a levitated nanoparticle via coherent scattering. Phys. Rev. Lett..

[57] de los Ríos Sommer A, Meyer N, Quidant R (2021). Strong optomechanical coupling at room temperature by coherent scattering. Nat. Commun..

[58] Dare, K. et al. Linear ultrastrong optomechanical interaction. Preprint at arXiv:2305.16226 [quant-ph] (2023).

[59] Wilson-Rae I, Nooshi N, Dobrindt J, Kippenberg TJ, Zwerger W (2008). Cavity-assisted backaction cooling of mechanical resonators. J. Phys..

[60] Gonzalez-Ballestero, C. Tutorial: projector approach to open quantum systems. Preprint at arXiv:2305.19704

